# Assessing Family Medicine Residents’ Knowledge, Attitudes, and Confidence in Managing In-Flight Medical Emergencies in Riyadh, Saudi Arabia

**DOI:** 10.7759/cureus.46620

**Published:** 2023-10-07

**Authors:** Abdulaziz S Alarifi, Norah ALRowais

**Affiliations:** 1 Family and Community Medicine, King Saud University Medical City, Riyadh, SAU

**Keywords:** riyadh, family medicine residents, medical emergencies, in-flight, confidence

## Abstract

The global increase in air travel has led to a rise in in-flight medical emergencies (IMEs), posing significant challenges to global public health. In a significant number of instances, physicians are often called upon to respond to IMEs during flights. These emergencies are unique due to the cabin environment's constraints, including confined space, ambient noise, and reduced atmospheric pressure. Various proactive measures have been introduced to mitigate IME occurrences, but many healthcare professionals, including family medicine (FM) residents, feel inadequately prepared to respond effectively.

This cross-sectional study was conducted among all family medicine (FM) residents in Riyadh, Saudi Arabia, in January 2023. A self-reported questionnaire was used, including questions aimed at evaluating the sociodemographics, travel profiles, knowledge, confidence, and attitudes of FM residents toward IMEs and their ability to respond to such emergencies.

A total of 189 FM residents participated in the study, revealing a young and diverse group of participants. Most were male (97 (51.3%)), averaging 27 years old. Nearly all held life support credentials (185 (97.9%)), particularly basic life support (BLS) and advanced cardiovascular life support (ACLS). Despite frequent travel, the majority lacked in-flight emergency training and experience. Allergic reactions (28%), cardiovascular (24%), and respiratory emergencies (24%) were the most common IMEs encountered.

While 109 (57.7%) would assist during an IME, 87 (46%) were unsure of their competence, and 109 (57.7%) had medico-legal concerns. Most, i.e., 176 (93.2%) agreed with the need for more IME training, but 138 (73%) lacked clarity about in-flight medical supplies. In conclusion, this research underscores the importance of preparing FM residents and healthcare professionals for IMEs, advocating for specialized training programs that enhance their readiness to respond competently to an IME.

## Introduction

Over the past few decades, more individuals have traveled by air daily, both domestically and internationally [1]. This rise in air travel has led to an increased occurrence of in-flight medical emergencies (IMEs), imparting substantial implications for global and public health [1]. Cabin crew often seek medical assistance, utilizing onboard medical kits, diverting flights, and relying on ground-based support to manage such emergencies [1].

Notably, it is not unusual for medically trained individuals, including physicians, to find themselves facing such high-stress scenarios during a flight. Research indicates that physicians voluntarily step forward to assist in 43% to 85% of reported IMEs, a situation that can be demanding even for seasoned medical professionals [2,3]. In-flight medical emergencies lack a clear and universally accepted definition. However, between 2008 and 2010, the estimated global incidence of IMEs stood at one occurrence per 604 flights [2]. The spectrum of emergencies experienced during flights encompasses a range of medical conditions, including gastrointestinal symptoms (e.g., nausea and vomiting), neurological manifestations (e.g., seizures, syncope), and cardiac events (e.g., acute exacerbations, chest pain) [2,4,5].

Physicians who respond to IMEs onboard have noted that the assessment and management of these emergencies differ from those occurring on the ground due to the unique cabin environment. Factors such as the confined space, ambient noise, and reduced atmospheric pressure necessitate adaptations in the delivery of medical care [6,7]. To mitigate the occurrence of IMEs, various proactive measures have been implemented, such as passenger prescreening for medical conditions that may lead to emergencies, medical training for cabin crew, online medical advice services, and the presence of in-flight volunteer medical staff [8-10]. However, a study conducted by Udezi et al. revealed that many participants feel inadequately prepared to prevent and respond to IMEs, with 72% of participants expressing a desire for more comprehensive training in this area [5].

The increasing frequency of IMEs presents an opportunity for family medicine (FM) residents, who are exposed to a wide range of multidisciplinary cases, to acquire the knowledge and skills necessary to counsel at-risk patients before their flights and respond to emergencies with confidence. A study conducted in the Kingdom of Saudi Arabia underscores the importance of IME training programs for all physicians, particularly those specializing in internal medicine and FM [11].

The literature emphasizes the need for comprehensive IME training programs to instill confidence and address knowledge gaps among future healthcare practitioners. Additionally, poor understanding of various IME-related aspects highlights the need for improved education and awareness in this critical area of medicine [5,11].

We believe that the FM residency program provides an ideal setting for such training, ultimately benefiting communities when IMEs arise. This study aims to evaluate the knowledge, confidence, and attitudes of FM residents regarding IMEs and their ability to respond effectively.

## Materials and methods

Study approval and ethical considerations

This study was approved by the Institutional Review Board Committee of King Saud University, ensuring adherence to ethical guidelines (approval no. E-22-7310). The confidentiality of participants was guaranteed. Written informed consent was obtained from all participants.

Study design

This research employed a quantitative, observational cross-sectional study design, conducted in January 2023. The study focused on FM residents in Riyadh, Saudi Arabia, and included both male and female participants enrolled in residency programs at different levels, including postgraduate year (PGY)-1, PGY-2, and PGY-3. Based on the Krejcie and Morgan sample size calculation formula for a finite population, the minimum recommended sample size was 186 residents. The survey was distributed to all FM residents, with a total of 362 respondents across eight programs in Riyadh, namely King Faisal Specialist Hospital and Research Center, Security Forces Hospital, King Abdullah bin Abdulaziz University Hospital, Prince Sultan Military Medical City (PSMMC), Riyadh Health Cluster 1, King Saud University Medical City (KKUH), National Guard Hospital, Riyadh Health Cluster 2.

Data collection

Data were collected using a self-administered questionnaire. The questionnaire was developed based on a comprehensive literature review. It comprised two parts: (1) participants' demographic characteristics and their exposure to in-flight medical emergencies; and (2) an assessment of participants' knowledge and confidence in managing these emergencies, drawing inspiration from the studies conducted by Katzer et al. and Ng et al. [3,7]. Participants received the questionnaire via an online Google Form (Alphabet Inc., Mountain View, CA, USA) link provided to their registered emails in the Saudi Commission for Health Specialties (SCFHS).

Data analysis

Descriptive statistics were used to summarize continuous data as mean (standard deviation) and dichotomous data as frequency (percentage). The normality of the results was assessed using the Kolmogorov-Smirnov test. A bivariate Pearson's correlation test was employed to examine correlations between measured metric variables. To determine the predictors of physicians' odds of having provided care for IMEs, a multivariable logistic binary regression analysis (MLBR) was performed. The results were presented as odds ratios (OR) with a 95% confidence interval (CI). The level of significance was set at 0.05. The statistical analysis was performed using SPSS Statistics version 21 (IBM Corp., Armonk, NY, USA).

## Results

The study included a total of 189 FM residents who completed the survey with a 52.2% response rate. The slight majority of respondents, 97 (51.3%), were male, with a mean age of 27 years. In terms of training status, 71 (37.6%) of the residents were in their final year of training (PGY-3), followed by PGY-2 with 67 (35.4%) residents and PGY-1 with 51 (27%) residents. A significant proportion (48 (25.4%)) of participants were undergoing residency training in Riyadh Health Cluster 2, as shown in Table 1.

**Table 1:** Sociodemographic characteristics of participants The continuous data is represented as frequency (percentage), and the dichotomous data is represented as mean (standard deviation). PGY: Postgraduate year

Characteristics		Frequency (percentage), N=189
Gender		
	Male	97 (51.3%)
	Female	92 (48.7%)
Age, mean (standard deviation)		27.25 (2.58)
Medical residency level/ rank		
	PGY-1	51 (27%)
	PGY-2	67 (35.4%)
	PGY-3	71 (37.6%)
Medical affiliation		
	Riyadh Health Cluster 2	48 (25.4%)
	National Guard Hospital	44 (23.3%)
	King Saud University Medical City (KKUH)	36 (19%)
	Prince Sultan Military Medical City (PSMMC)	24 (12.7%)
	Riyadh Health Cluster 1	24 (12.7%)
	King Abdullah bin Abdulaziz University Hospital	6 (3.2%)
	Security Forces Hospital	4 (2.1%)
	King Faisal Specialist Hospital & Research Center	3 (1.6%)

The demographic responses revealed that nearly all respondents (185 (97.9%)), held one or more life support credentials. Basic life support (BLS) and advanced cardiovascular life support (ACLS) were the most common certifications, held by 182 (98.9%) and 108 (58.7%) participants, respectively. Despite a substantial number (77 (40.7%)) of residents traveling frequently two or three times per year, with 45 (23.8%) traveling more than three times per year, the majority (178 (94.2%)) had not received any in-flight emergency course, 160 (84.7%) had not encountered any IMEs so far, and 161 (85.2%) had not provided medical assistance for an IME. Most participants (135 (71.4%)) believed that IME training should be mandatory for all medical specialties. As shown in Table 2, analysis of the data revealed that the most commonly encountered in-flight emergency conditions by participants were allergic reactions (28%), followed by cardiovascular (24%) and respiratory emergencies (24%).

**Table 2:** Perceptions and experiences of the participants The continuous data is represented as frequency (percentage).

Questionnaire	Frequency (Percentage)
Have you ever received any training course in the management of in-flight medical emergencies?	
No	178 (94.2%)
Yes	11 (5.8%)
Have you ever encountered any in-flight medical emergency before?	
No	160 (84.7%)
Yes	29 (15.3%)
What was/were the inflight medical emergency condition/s?	
Allergic	7 (28%)
Cardiovascular	6 (24%)
Respiratory	6 (24%)
Neurological	5 (20%)
Gastrointestinal	4 (16%)
Psychological	3 (12%)
Obstetric	2 (8%)
Diabetic	1 (4%)
Have you provided medical assistance for the in-flight affected person/s?	
No	161 (85.2%)
Yes	28 (14.8%)
In your opinion, training on an in-flight medical emergency has to be covered in which medical specialties?	
All specialties	135 (71.4%)
Emergency Medicine	56 (29.6%)
Family medicine	39 (20.6%)
Internal medicine	22 (11.6%)
Pediatrics	16 (8.5%)
Obstetrics and Gynecology	13 (6.9%)
Surgical specialties	4 (2.1%)
Are you certified with any life support credentials?	
No	4 (2.1%)
Yes	185 (97.9%)
What life support training do you have? (N=185)	
Basic life support (BLS),	182 (98.9%)
Advanced cardiovascular life support (ACLS)	108 (58.7%)
Advanced trauma life support (ATLS)	3 (1.6%)
Pediatric advanced life support (PALS)	2 (1.1%)
How often do you travel via airplane in a year?	
Never	1 (0.5%)
Once per year	66 (34.9%)
2-3 times per year	77 (40.7%)
>3 times per year	45 (23.8%)

Regarding their attitudes toward IMEs, the majority (109 (57.7%)() of participants would identify themselves as doctors and offer assistance if they experienced an IME. Approximately 87 (46%) would refrain from intervening if another healthcare provider offered help. However, about half of the participants (100 (52.9%)) expressed their willingness to offer assistance if they were the only healthcare provider on board, even if they were not familiar with the emergency. Additionally, 87 (46%) of participants were unsure about their confidence to respond to IMEs and provide competent care, and 68 (36%) lacked confidence in their abilities. Fear of potential medicolegal implications was a concern for more than half of the participants (109, (57.7%)). Furthermore, 77 (40.7%) admitted that their medical training had not adequately equipped them with the knowledge and skills to manage IMEs. The majority (176 (93.2%)) agreed on the need for more training in managing IMEs. Furthermore, as shown in Table 3, 138 (73%) participants reported not having a clear understanding of the available medical supplies on commercial airplanes, 124 (65.9%) had the level of training of commercial aircrew in managing IMEs, and 109 (57.7%) had the manner of collaboration among aircrew, ground-based medical control, and onboard volunteer healthcare providers.

**Table 3:** Attitudes and confidence of the participants toward IMEs The continuous data is represented as frequency (percentage), and the dichotomous data is represented as mean (standard deviation). IMEs: In-flight medical emergencies

Statements	Mean (SD)	Disagree/Strongly Disagree N (%)	Undecided, N (%)	Agree/Strongly Agree, N (%)
I would identify myself as a doctor and offer assistance in the event of an in-flight medical emergency.	3.67 (0.97)	22 (11.6%)	58 (30.7%)	109 (57.7%)
I would stay out of an in-flight medical emergency if there is already someone else offering their assistance.	3.32 (1.03)	36 (19%)	66 (34.9%)	87 (46%)
I would not offer assistance if I am not familiar with the nature of the emergency, even though I am the only healthcare professional onboard.	2.62 (1.07)	100 (52.9%)	47 (24.9%)	42 (22.2%)
I am afraid of the medicolegal implications that may arise from my assistance in an in-flight medical emergency.	3.58 (1.12)	34 (18%)	46 (24.3%)	109 (57.7%)
I need more training in managing in-flight medical emergencies.	4.52 (0.64)	1 (0.5%)	12 (6.3%)	176 (93.2%)
My medical training has given me adequate knowledge and skills to render assistance during an in-flight medical emergency.	2.76 (1.01)	77 (40.7%)	74 (39.2%)	38 (20.1%)
I would currently feel confident responding to an in-flight medical emergency and providing competent care.	2.78 (0.90)	68 (36%)	87 (46%)	34 (18%)
I have an adequate understanding of what medical supplies are available on commercial airplanes.	2.04 (0.97)	138 (73%)	36 (19%)	15 (7.9%)
I have an adequate understanding of the level of training of commercial aircrew in managing in-flight medical emergencies.	2.21 (0.94)	124 (65.9%)	49 (25.9%)	16 (8.5%)
I have an adequate understanding of the manner in which the aircrew, ground-based medical control, and the onboard volunteer healthcare provider collaborate to manage an in-flight medical emergency.	2.35 (0.98)	109 (57.7%)	59 (31.2%)	21 (11.1%)

Our multivariate logistic regression analysis revealed significant associations between various factors and the likelihood of participants offering medical care for IMEs. Specifically, participants who traveled two or more times per year exhibited significantly higher odds of providing medical care for IMEs (OR = 2.108, p = 0.015), and similarly for those having a high mean of perceived confidence to intervene in an in-flight medical emergency (OR = 3.530, p = 0.001). In contrast, no significant relationship was found between offering medical care for IMEs and gender (OR = 0.605, p = 0.292), age (OR = 1.039, p = 0.711), level of training (OR = 1.097, p = 0.762), and prior receipt of IMEs training (OR = 2.497, p = 0.234) (see Table 4).

**Table 4:** Multivariable analysis of the participants' odds of providing care in IMEs The level of significance was set at p = 0.05 IMEs: In-flight medical emergencies, OR: Odds Ratio, CI: Confidence Interval

Variable	Adjusted (OR)	95% CI	p-value
Gender (male)	0.605	(0.238-1.540)	0.292
Age (years)	1.039	(0.848-1.272)	0.711
Level of training	1.097	(0.602-2.001)	0.762
Frequency of flight travel ≥ 2 times/year	2.108	(1.157-3.839)	0.015
Mean of perceived confidence to intervene with an in-flight medical emergency	3.530	(1.689-7.376)	0.001
Had previously received flight medical emergency training	2.497	(0.554-11.260)	0.234

## Discussion

Our study, involving 189 family medicine residents, offers a comprehensive view of the demographics and training backgrounds of this crucial healthcare professional group. The gender distribution in our sample, with a majority of male participants at 97 (51.3%), mirrors the broader gender distribution trends observed in medical fields, although the gender gap in medicine is narrowing over time [1,6,12]. 

Furthermore, the relatively young age of our respondents, with a mean age of 27 years, reflects the early stages of their medical careers. This demographic characteristic underscores the importance of providing comprehensive training and support to individuals who are embarking on their journey in the medical profession, as they represent the future of healthcare delivery [2]. Our study also revealed the distribution of residents across different training years, highlighting the diverse experience levels within the study population [2]. This diversity is critical when considering the preparedness of family medicine residents to respond to medical emergencies, as those in more advanced training years may have had more exposure to clinical situations and may be more confident in their abilities.

One striking observation from our study is the high percentage of residents (185, (97.9%)) holding one or more life support credentials, particularly BLS and ACLS. This finding emphasizes the dedication of FM residents to their professional development and preparedness to manage critical situations, which is consistent with previous literature emphasizing the importance of healthcare providers' readiness to respond to IMEs [1,3,7,13].

However, a significant concern arises from the fact that a substantial proportion of participants frequently traveled by air but had not received formal in-flight emergency training. This discrepancy underscores a potential gap in their education and readiness to respond effectively to IMEs in aviation settings. While BLS and ACLS certification are essential, IMEs during air travel present unique challenges and require specialized training to address the specific conditions and constraints of in-flight medical care [2,7].

Our research findings shed light on the prevalence of specific medical issues during air travel. Notably, allergic reactions, cardiovascular events, and respiratory emergencies were identified as the most common IMEs encountered. These results align with previous research, emphasizing the importance of preparedness for these scenarios, especially considering that physicians are often called upon to assist with in-flight emergencies [1,8,11].

An intriguing aspect of our study is the mixed attitudes of FM residents toward IMEs. While a significant portion expressed a willingness to offer assistance if they were the sole healthcare provider onboard, a substantial number were uncertain about their ability to respond effectively. Fear of medicolegal implications emerged as a prevalent concern, corroborating findings from similar studies [4,11]. These attitudes highlight the need for targeted training programs that not only enhance the clinical skills of FM residents but also address their concerns about potential legal repercussions when providing medical assistance in aviation contexts [4,8].

An overwhelming finding from our study is the recognition among participants that additional training is needed, with 176 (93.2%) expressing this sentiment. This underscores the urgency of enhancing the preparedness of FM residents for IMEs and serves as a compelling call to action for medical training programs to address these concerns comprehensively [5].

Our research also unveiled a lack of understanding among participants regarding various IME-related aspects, including the available medical supplies on commercial airplanes, the level of training of commercial aircrew in managing IMEs, and the manner of collaboration among aircrew, ground-based medical control, and on-board volunteer healthcare providers. These findings accentuate the need for improved education and awareness among healthcare professionals regarding the intricacies of in-flight medical care, aligning with the call for specialized training programs [5,8,11].

This study, to the best of our knowledge, is the first of its kind to investigate the readiness of FM residents in Saudi Arabia to respond to IMEs. One limitation is the small sample size, which makes comprehensive comparisons challenging due to the limited relevant studies in the literature. Future research should focus on robustly validating the knowledge questionnaire and including a larger and more diverse group of physicians for broader insights.

Despite these limitations, the findings provide a vital starting point for future investigations. They highlight the necessity for further research in FM and similar residency programs concerning IME education. Moreover, there is an opportunity to enhance medical training curricula by integrating IMEs as a crucial component, ultimately leading to better-prepared physicians and improved passenger safety during air travel.

## Conclusions

Our study highlights the need for specialized training programs to prepare FM residents for IMEs. While many residents hold life support credentials, a gap exists in formal in-flight emergency training, which is crucial for addressing unique challenges during air travel. Common IME scenarios include allergic reactions, cardiovascular events, and respiratory emergencies. Attitudes toward IMEs vary, with some residents willing to assist while others express doubts and legal concerns. The majority agree that additional training is necessary. Furthermore, participants lack understanding of key IME aspects, such as available medical supplies on airplanes and collaboration between aircrew and medical personnel.

To address these findings, we recommend the development of specialized IME training modules that cover clinical skills and legal aspects. Raising awareness about in-flight medical care intricacies is also essential. These measures will better prepare healthcare practitioners and enhance passenger safety during air travel.
